# Fractional Coupling of Primary and Johari–Goldstein Relaxations in a Model Polymer

**DOI:** 10.3390/polym14245560

**Published:** 2022-12-19

**Authors:** Carlo Andrea Massa, Francesco Puosi, Dino Leporini

**Affiliations:** 1 Istituto per i Processi Chimico-Fisici-Consiglio Nazionale delle Ricerche (IPCF-CNR), Via G Moruzzi 1, 56124 Pisa, Italy; 2 Istituto Nazionale di Fisica Nucleare, Largo B. Pontecorvo 3, 56127 Pisa, Italy; 3 Dipartimento di Fisica ‘Enrico Fermi’, Università di Pisa, Largo B. Pontecorvo 3, 56127 Pisa, Italy

**Keywords:** polymer melt, Johari-Goldstein relaxation, dynamic heterogeneity, molecular-dynamics simulation

## Abstract

A polymer model exhibiting heterogeneous Johari–Goldstein (JG) secondary relaxation is studied by extensive molecular-dynamics simulations of states with different temperature and pressure. Time–temperature–pressure superposition of the primary (segmental) relaxation is evidenced. The time scales of the primary and the JG relaxations are found to be highly correlated according to a power law. The finding agrees with key predictions of the Coupling Model (CM) accounting for the decay in a correlation function due to the relaxation and diffusion of interacting systems. Nonetheless, the exponent of the power law, even if it is found in the range predicted by CM (0<ξ<1), deviates from the expected one. It is suggested that the deviation could depend on the particular relaxation process involved in the correlation function and the heterogeneity of the JG process.

## 1. Introduction

By lowering the temperature *T* or increasing the pressure *P* and avoiding crystallization, polymeric dense melts transform into a glass [1]. Close to the glass transition, relaxation occurs via both the primary (α) and the faster secondary (β) processes [2,3,4]. The β process has been intensively studied over the years [4,5,6,7,8,9,10].

In linear polymers, the secondary relaxation is due to the dynamics of the fragments of the chain [2,11,12,13] and considered a genuine manifestation of the Johari–Goldstein (JG) β relaxation [5]. The close relationship of the JG relaxation with the α relaxation has been noted [4,6,7,8,14], also due to the fact that both of them exhibit broad distribution of relaxation times [3,15,16] and cooperative dynamics [8,17,18].

It was early noted that the molecular reorganisation giving rise to the JG relaxation process is similar to those involved in the glass transition itself [19] with extensive later support [4,6,7,13,20,21,22,23]. It was concluded that the JG relaxation is a precursor to structural relaxation and viscous flow, with sluggish dynamics due to cooperativity driven by many body dynamics [6,8,15,18,24].

The several common features between JG and primary relaxation suggests that the secondary relaxation time $\tau_{\beta }$ and the primary relaxation time $\tau_{\alpha }$ are correlated. This aspect has been widely investigated by the Coupling Model (CM) developed by Ngai and coworkers, who predicted the many-body effects in relaxation and diffusion of interacting systems [4]. CM focusses on the independent or primitive relaxation with time scale $\tau_{0}$. At times shorter than $t_{c}$ ($t_{c}≃2$ ps, insensitive to both the temperature *T* and the pressure *P*), the basic molecular units relax independently of each other and the correlation function ϕ(t) relaxes as an exponential with decay time $\tau_{0}$:

(1) $$\phi \left(t\right)=\exp\left(-\frac{t}{\tau_{0}}\right)\;t<t_{c}$$

For $t>t_{c}$, the intermolecular interactions slow down the α relaxation and the correlation function assumes the Kohlrausch–Williams–Watts (KWW) stretched exponential form (0<β≤1):

(2) $$\phi^{kww}\left(t\right)=\exp\left[-{\left(\frac{t}{\tau }\right)}^{\beta }\right]\;t>t_{c}$$

with $\tau =\tau_{\alpha }$. The primitive relaxation is considered as precursor of the α relaxation, as expressed by the relation between their respective time scales:

(3) $$\tau_{0}={\left(t_{c}\right)}^{n}{\left(\tau_{\alpha }\right)}^{\beta }$$

where β=1−n. Equation (3) follows from the requirement of continuity of ϕ(t), as given by Equations (1) and (2), at $t_{c}$ and holds in the limit $t_{c}\ll \tau_{0}$ [4]. In many glass formers with genuine JG relaxations, it is found that, on changing both *T* and *P*, $\tau_{0}\left(T,P\right)≃\tau_{\beta }\left(T,P\right)$ [7]. Then, Equation (3) is finally recast as

(4) $$\tau_{\beta }={\left(t_{c}\right)}^{n}{\left(\tau_{\alpha }\right)}^{\beta }$$

Noticeably, Equation (4) predicts a power–law relation between $\tau_{\beta }$ and $\tau_{\alpha }$ if the exponent β is *constant, i.e., it does not depend on T* and *P*. Given the generic form of the correlation function ϕ(t) assumed by CM, this conclusion has to be considered as independent of the particular relaxation process considered by ϕ(t). Equation (4) suggests that the JG relaxation and the primary relaxation are strongly correlated due to the intermolecular interactions whose influence becomes apparent for times exceeding $t_{c}$, a few picoseconds, i.e., much shorter than both $\tau_{\beta }$ and $\tau_{\alpha }$.

Close to the glass-transition spatial correlations between dynamic fluctuations—so-called dynamic heterogeneity (DH)—become apparent during the evolution of the α process [25,26,27,28,29,30,31,32,33]. Nonetheless, DH has been evidenced from picosecond [9] through β relaxation time scales [34,35,36], in agreement with previous suggestions [22].

The usual tool to characterize DH is the non-Gaussian parameter (NGP) [37]:

(5) $$\alpha_{2}\left(t\right)=\frac{3}{5}\frac{\langle r^{4}\left(t\right)\rangle }{{\langle r^{2}\left(t\right)\rangle }^{2}}-1$$

where r(t) is the modulus of the particle displacement in a time *t*. Brackets denote the ensemble average. NGP vanishes if the displacement is a spatially homogeneous single Gaussian-random process [38]. NGP has recently revealed the JG relaxation in metallic glasses [24] and polymers [36].

In this work, we report results from thorough molecular-dynamics (MD) simulations of a polymer model melt exhibiting strong DH of both the primary and JG relaxations, and *constant* stretching parameter β of the primary relaxation. Evidence is given of a power-law correlation between $\tau_{\beta }$ and $\tau_{\alpha }$. The finding is consistent with Equation (4). However, the exponent of the power law is found to be different from β.

## 2. Model and Numerical Methods

A dense melt of coarse-grained (bead-spring) linear polymer chains with $N_{c}=512$ linear chains made of M=25 monomers with mass *m* each, results in a total number of monomers *N* = 12,800 being studied by MD simulations [36]. Non-adjacent monomers in the same chain or monomers belonging to different chains are defined as “non-bonded” monomers. Non-bonded monomers at mutual distance *r* interact via a shifted Lennard–Jones (LJ) potential:

(6) $$U^{LJ}\left(r\right)=\epsilon \left[{\left(\frac{\sigma^{*}}{r}\right)}^{12}-2{\left(\frac{\sigma^{*}}{r}\right)}^{6}\right]+U_{cut},$$

$\sigma^{*}=2^{1/6}\sigma$ is the minimum of the potential, $U^{LJ}\left(r=\sigma^{*}\right)=-\epsilon +U_{cut}$. The potential is truncated at $r=r_{c}=2.5\sigma$ and the constant $U_{cut}$ adjusted to ensure that $U^{LJ}\left(r\right)$ is continuous at $r=r_{c}$ with $U^{LJ}\left(r\right)=0$ for $r\geq r_{c}$. Along the same linear chain, monomers are bonded by the harmonic potential $U^{bond}\left(r\right)=k_{bond}{\left(r-l_{0}\right)}^{2}$, where the constant $k_{bond}=2000\epsilon /\sigma^{2}$ to ensure high stiffness and the rest of the bond length $l_{0}=0.48\sigma$. A bending potential $U^{bend}\left(\alpha \right)=k_{bend}{\left(\cos\alpha -\cos\alpha_{0}\right)}^{2}$ ($k_{bend}=2000\epsilon ,\alpha_{0}=120^{\circ }$) ensures that the angle α between two consecutive bonds is nearly constant. The above model builds a torsional barrier when $l_{0}<0.5\sigma$—as in the present work—which is discussed elsewhere [36]. Our chain model exhibits a significant local stiffness. In fact, the length $\ell_{K}$ of the associated Kuhn segment [39] is $\ell_{K}$∼2, larger than the one of flexible polymer model, such as the Kremer–Grest model with $\ell_{K}^{KG}$∼1 [40].

All the data presented in the work are expressed in reduced MD units: length in units of σ; temperature in units of $\epsilon /k_{B}$, where $k_{B}$ is the Boltzmann constant; and time in units of $\tau_{MD}={\left(m\sigma^{2}/\epsilon \right)}^{1/2}$. Roughly, $\tau_{MD}=1$ corresponds to about 1 ps [41]. We set σ=1, ϵ=1, m=1 and $k_{B}=1$.

Simulations were carried out with the open-source software LAMMPS [42,43]. Equilibration runs were performed at constant pressure *P* and temperature *T* (NPT ensemble) [44] (details about the barostat are found elsewhere [42,43]; the barostat damping parameter equals to 0.1 in MD time units). The investigated (P,T) pairs are listed in Table 1. It is worth nothing that, although some physical states (P=0;T=0.85,0.9,0.95,1.1) were also considered elsewhere [36], the data reported in the present work have been produced independently, following the protocol mentioned above, so as to ensure maximum consistency and further support their robustness.

For each state, the equilibration was terminated not earlier than three times the end-to-end relaxation time [39]. Production runs were performed within the NVT ensemble [44]. Pressure was evaluated during all the production runs to monitor the full consistency with the pre-set value of the NPT equilibration run. Other details are given elsewhere [36].

## 3. Results

### 3.1. Bond Correlation Function

It has been demonstrated that the reorientation processes exhibit particular sensitivity to secondary motions [35,45,46,47,48]. From this respect, a convenient process is the reorientation of the chain bonds [35,48] with bond correlation function (BCF) [49]:

(7) C(t)=〈cosθ(t)〉

θ(t) is the angle spanned in a time *t* by the unit vector along a generic bond of a chain.

**Figure 1:**
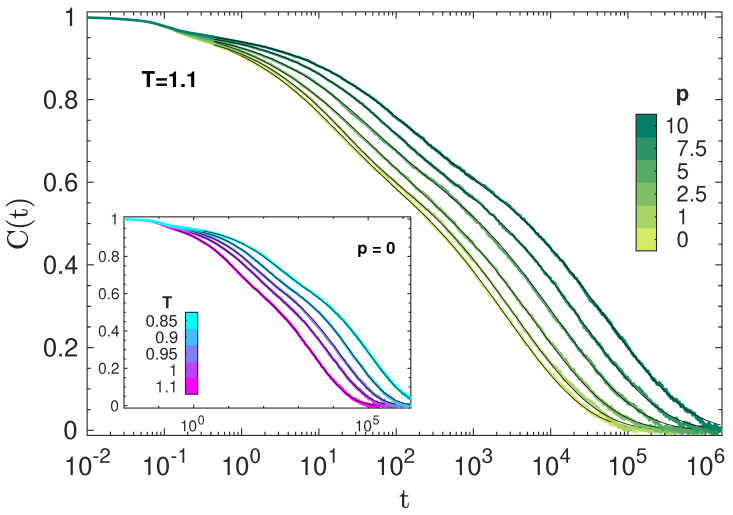
Isothermal and isobaric (inset) plots of selected BCFs, Equation (7). The superimposed thin solid black lines are the best fit with Equation (8).

Figure 1 plots representative isothermal and isobaric decays of BCF. It exhibits an initial decay for t≲1 which is virtually independent of (P,T). Then, a characteristic two-step drop is apparent, evidencing two relaxation processes, i.e., the fast JG and the slow primary (segmental) relaxations [36,48,50,51].

### 3.2. Time Temperature and Pressure Superposition of Primary Relaxation

**Figure 2:**
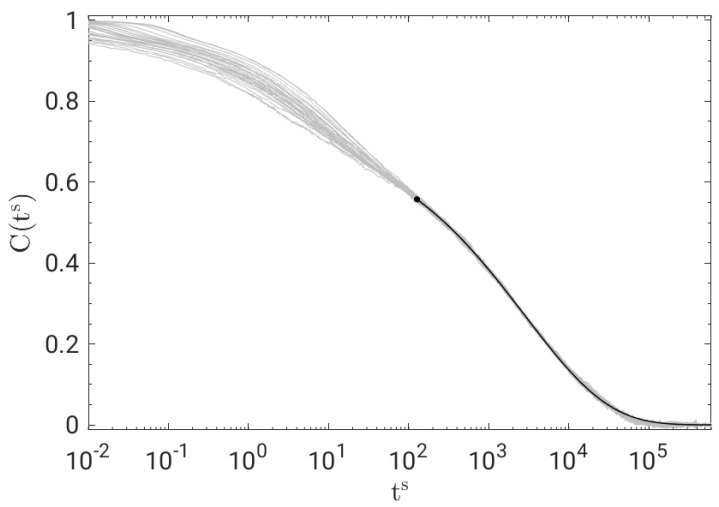
Time−temperature−pressure superposition (TTPS) of BCF for all the states in Figure 1. The curves are shifted along the horizontal axis to optimise their superposition at long times. The superimposed solid black line is the best fit with a stretched exponential proportional to $\phi^{kww}\left(t\right)$, Equation (2), with $\beta_{\mathrm{TTPS}}=0.415$.

At *long* times, the shape of the BCF decay does not depend on the physical state. To show that, Figure 2 plots the master curve obtained by shifting the curves of Figure 1 horizontally, resulting in a remarkable time–temperature–pressure superposition (TTPS) in the time window of primary relaxation for $\mathrm{BCF}(t^{s})≲0.55$. The best fit of the master curve with the stretched exponential $A\phi^{kww}\left(t\right)$, where *A* is an adjustable constant, yields $\beta_{\mathrm{TTPS}}=0.415$.

TTPS motivated us to fit the whole BCF decay using different fit functions ensuring decay with *constant* stretching at long times. First, we adopted a weighted sum of two stretched exponentials, accounting for the primary (p) and the secondary (s) JG relaxation:

(8) $$C\left(t\right)=\Delta_{p}\phi_{p}^{kww}\left(t\right)+\Delta_{s}\phi_{s}^{kww}\left(t\right)$$

where $\phi_{i}^{kww}\left(t\right)$, i=p,s is Equation (2) with $\tau =\tau_{i}$, $\beta =\beta_{i}$. We set $\beta_{p}=\beta_{\mathrm{TTPS}}=0.415$ so as to leave Equation (8) with five adjustable parameters, i.e., $\Delta_{p},\Delta_{s},\tau_{p},\tau_{s},\beta_{s}$. The best-fit procedure yields excellent agreement, as shown in Figure 1.

**Figure 3:**
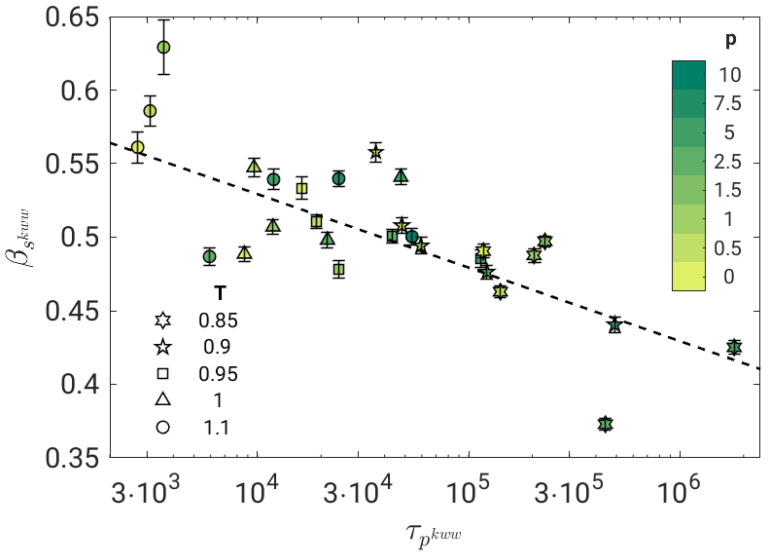
Stretching parameter of the secondary JG relaxation of all the investigated states according to the best fit with Equation (8). The dashed line is a guide for the eyes. Stretching increases mildly with the primary relaxation time.

Figure 2 shows that at *short* times in the JG time window, TTPS does not work. In fact, the best-fit procedure performed with Equation (8) returns stretching parameters of the JG relaxation which depend on the state and tend to decrease by slowing down the primary relaxation, see Figure 3.

### 3.3. Dynamic Heterogeneity

**Figure 4:**
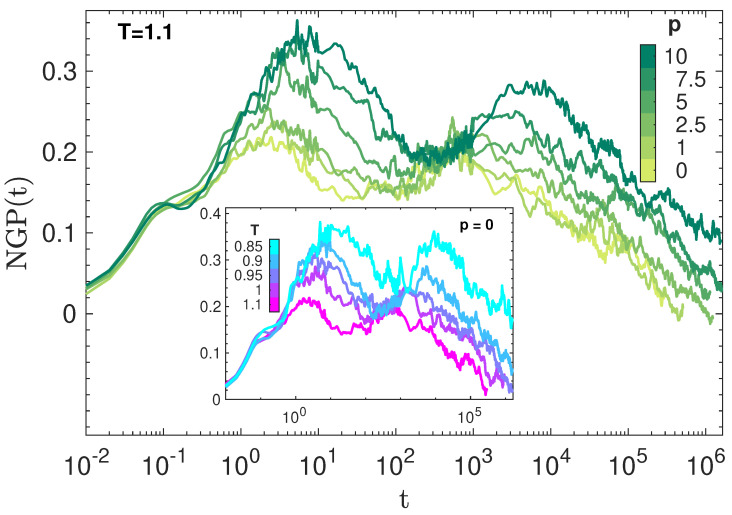
NGP, Equation (5), of all the states in Figure 1. DH is apparent in both the secondary JG relaxation time scale, *t*∼1–10, and the the primary, segmental relaxation, *t*∼$10^{3}$–$10^{4}$.

Figure 4 plots the NGP of the states in Figure 1 and shows how their DH develops with time. As in other studies, NGP is largely independent of (P,T) at very short times (t≲0.1), suggesting a major role of static structure [35]. The small peak at about 0.1 marks the average time between two consecutive collisions of the monomer with the cage formed by the closest neighbours [52]. For t>0.1, NGP is strongly dependent on (P,T). Two peaks are observed, the one occurring at a shorter time attributed to the JG heterogeneity whereas the one located at longer times are due to the familiar DH of the primary, structural relaxation [36]. The height of the NGP parameter in the JG region is not surprising, given the considerable stretching of the JG relaxation seen in Figure 3.

### 3.4. Fractional Coupling of Primary and JG Relaxations

**Figure 5:**
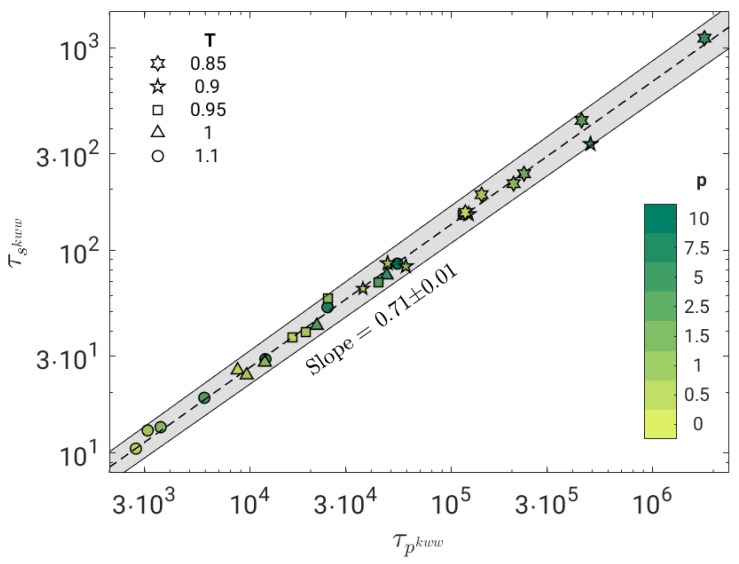
Correlation plot between the JG relaxation time $\tau_{s}$ and the primary relaxation time $\tau_{p}$ of all the investigated states, as drawn by fitting BCF C(t) with a weighed superposition of two stretched exponentials, Equation (8). The correlation is excellent (Pearson correlation coefficient R=0.998) and best fit with a power law with slope ξ=0.71±0.01 (dashed line). The grey area is the confidence region within one standard deviation of the best-fit parameters.

We now test the possible correlation between the time scales of the primary, $\tau_{p}$, and the secondary JG, $\tau_{s}$, relaxation processes. Figure 5 shows the result by fitting the BCF in terms of Equation (8). The correlation is excellent (Pearson correlation coefficient R=0.998) and well-expressed by a power law with exponent ξ=0.71±0.01.

To test the robustness of the result, we fit BCF with other functions with the same number of adjustable parameters. No significant changes were observed, i.e., $\tau_{p}$ and $\tau_{s}$ exhibit excellent power-law correlation with rather similar exponent. As an example, we considered the Williams ansatz [35,53,54,55,56]:

(9) $$C\left(t\right)=[\Delta_{p}+\Delta_{s}\phi_{s}^{kww}\left(t\right)]\phi_{p}^{kww}\left(t\right)$$

which leads to ξ=0.69±0.01 (R=0.997). Furthermore, following [16,35,55,56], we replaced $\phi_{s}^{kww}$ in Equation (8) with the Mittag–Leffler function:

(10) $$\phi_{s}^{ml}\left(t\right)=E_{a}\left(-\left(t/\tau_{s}\right)\right)$$

with

(11) $$E_{a}\left(x\right)=\sum_{k=0}^{\infty }\frac{x^{k}}{\Gamma \left(ak+1\right)}$$

where Γ(x) is the gamma function. This leads to ξ=0.69±0.01 (R=0.996). If the same replacement is performed in Equation (9), we find ξ=0.68±0.01 (R=0.996).

## 4. Discussion

The strong power-law correlation between the primary and the JG relaxation, evidenced by Figure 5, is the key result of the present work. The power-law coupling between different time scales in systems with significant DH is documented, a well-known example being the breakdown of the Stokes–Einstein relation [26,57].

The coupling model (CM) offers a highly investigated conceptual framework predicting, according to Equation (4), the fractional coupling between the JG and the primary relaxation. Notably, if TTPS holds, the stretching parameter β of the primary relaxation is independent of the (P,T) state and the fractional coupling reduces to a power law between $\tau_{\beta }$ and $\tau_{\alpha }$ with exponent β (0<β≤1). Indeed, the polymer model under study exhibits TTPS, Figure 2, and a power-law correlation between $\tau_{\beta }$ and $\tau_{\alpha }$ in the investigated (P,T) range, Figure 5, with exponent 0<ξ<1. These findings are fully consistent with CM. However, the exponent of the power law (ξ=0.71±0.01) differs from the stretching parameter of the primary relaxation ($\beta_{\mathrm{TTPS}}=0.415$).

To the present level of understanding, the disagreement is not easily interpreted. We offer some tentative routes to be explored in future studies. First, notably, while the well-known phenomenon of DH on the time scale of the primary (structural ) relaxation plays a central role in CM via the stretched relaxation, Equation (2), the influence of much less investigated DH on the JG time scale, which is evidenced in the present and previous studies [36], is not apparent in CM. Furthermore, we notice that CM is quite generic, i.e., the predictions are *independent* of the relaxation process involved in the correlation function ϕ(t). However, if the torsional autocorrelation function is studied by MD simulations of a polymer model which is rather similar to the present one, one finds a power law between JG and primary relaxation with an exponent $\xi_{BS}$∼0.25 (see Figure 7 in ref. [51]), which is almost three times less than ours (ξ=0.71±0.01) by considering BCF. This finding suggests that, even if the power-law coupling is robust, i.e., it is revealed by different correlation functions, and captured by CM, the exponent ξ of the power law and the stretching β could depend on the particular relaxation process in a different way. Unfortunately, no information about the possible TTPS and the stretching parameter of the primary relaxation is given in ref. [51], thus hampering a closer comparison with the present study. We noted elsewhere that BCF is more sensitive to the JG relaxation than the torsional autocorrelation function in the polymer model of the present study [48].

The influence of both the choice of the correlation function, as well as the magnitude of DH in JG relaxation, on the observation of the fractional coupling between the primary and the JG relaxation is postponed to future systematic studies.

## 5. Conclusions

A polymer model exhibiting heterogeneous JG secondary relaxation was studied by extensive MD simulations of states with different temperature and pressure. The TTPS of the primary (segmental) relaxation is evidenced. The time scales of the primary and the JG relaxations are found to be highly correlated according to a power law in agreement with CM predictions. Nonetheless, the exponent of the power law, even if it is in the CM range (0<ξ<1), deviates from the expected one. This motivates further investigation of the particular relaxation process involved in the correlation function addressed by CM and the heterogeneity of the JG process.

## Figures and Tables

**Table 1:** Investigated temperature and pressure values.

T∖p	0	0.5	1	1.5	2.5	5	7.5	10
1.1	${}^{\circ }$	${}^{\circ }$	${}^{\circ }$		${}^{\circ }$	${}^{\circ }$	${}^{\circ }$	${}^{\circ }$
1	${}^{\circ }$	${}^{\circ }$	${}^{\circ }$		${}^{\circ }$	${}^{\circ }$		
0.95	${}^{\circ }$	${}^{\circ }$	${}^{\circ }$		${}^{\circ }$	${}^{\circ }$		
0.9	${}^{\circ }$	${}^{\circ }$	${}^{\circ }$		${}^{\circ }$	${}^{\circ }$		
0.85	${}^{\circ }$	${}^{\circ }$	${}^{\circ }$	${}^{\circ }$	${}^{\circ }$	${}^{\circ }$		

## Data Availability

The data presented in this study are available on request from the corresponding author.

## Abbreviations

The following abbreviations are used in this manuscript:

BCF	Bond correlation function
CM	Coupling model
DH	Dynamical heterogeneity
JG	Johari–Goldstein
KWW	Kohlrausch–Williams–Watts
LJ	Lennard–Jones
MD	Molecular dynamics
NGP	Non-Gaussian parameter
NPT	Constant number of monomers *N*, constant pressure *P* and constant temperature *T*
NVT	Constant number of monomers *N*, constant volume *V* and constant temperature *T*
TTPS	Time–temperature–pressure superposition
